# Ultrasonography of an Oral Cavity Onchocercidae Nodule

**DOI:** 10.4269/ajtmh.17-0796

**Published:** 2018-03

**Authors:** Mattia Trunfio, Silvia Scabini, Roberto Bertucci

**Affiliations:** Unit of Infectious Diseases, Amedeo di Savoia Hospital, Department of Medical Sciences, University of Torino, Torino, Italy

A 51-year-old caucasian woman, living in Italy since her birth, presented with a 6-month history of a left swollen cheek and intraoral nodule, unresponsive to antibiotics. Her previous medical history was positive for seasonal allergies and left jowl reconstructive surgery with filling for a car accident 5 years before. Blood tests were unremarkable; physical examination showed an asymptomatic left cheek bulge and a 1 × 0.5 cm nontender noninflammatory submucosal firm nodule located in the left maxillary vestibule. An intraoral ultrasound examination was performed (Figure 1). Differential diagnoses of benign and malignant lesions were likely ruled out and preliminary diagnoses of probable filariasis or foreign body reaction were made. Filariasis serology and microfilaremia were negative. The patient refused antiparasitic treatment, and an excisional biopsy was performed; the healing was complicated by a surgical site infection. Microscopy revealed a cystic cavity containing a cross section of an onchocercidae worm (Figure 2). Considering the clinical picture, histology, and epidemiology, an ultimate presumable diagnosis of dirofilariasis was made.

**Figure 1.**
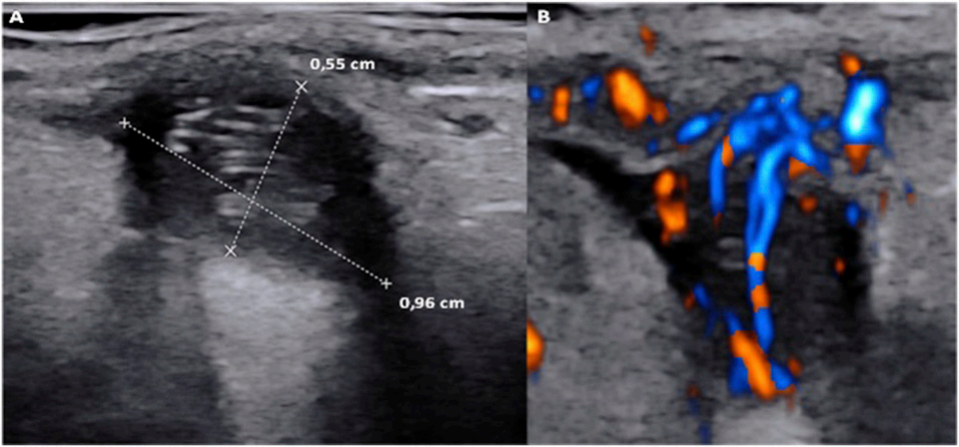
(**A**) A cystic lesion approximately of 1 cm in diameter, localized in the submucosa of the left superior maxillary vestibule, containing anechogenic fluid and a solid filiform mobile worm-like structure with echogenic posterior reinforcement (fluent weak movements were video recorded; see Supplemental Video 1). (**B**) Color doppler ultrasonography of the same cystic lesion showing suggestive twinkling artifact and no typical nor suspicious vascular pattern. This figure appears in color at www.ajtmh.org.

**Figure 2.**
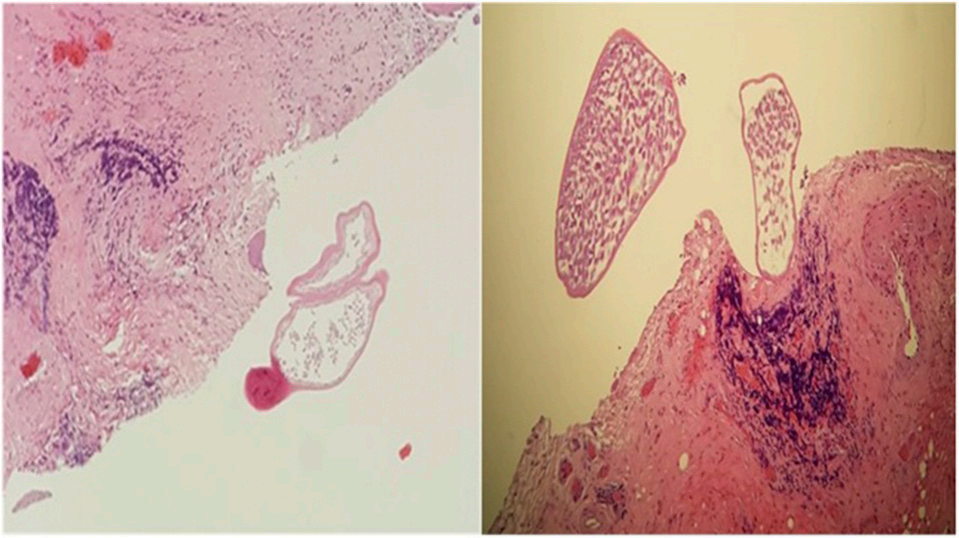
The histology demonstrates an abscess cavity lined with marked fibrosis and a moderate inflammatory response. No calcifications were observed. At the center of the cavity, the presence of disintegrating cross sections of an adult onchocercidae worm endowed with a characteristic muscle layer surrounded by a thick cuticle can be observed (H&E stain, 4x; courtesy of Pathology Unit, Maria Vittoria Hospital, Torino, Italy). This figure appears in color at www.ajtmh.org.

Dirofilaria are mosquito-borne nematodes infecting canines.^1^ Man is a dead-end host rarely infected by bites of vector mosquitoes.^1^ Endemic areas include tropical and subtropical regions, the United States, Australia, and the Mediterranean area.^1^ Recently, dirofilariasis spread to previously free countries because of climate changes and emerging vectors.^2^

Most of the cases involve the head, neck, or periocular region; oral dirofilariasis is rare.^3^ The most common presentation is a single symptomless subcutaneous/submucosal nodule.^3^ As in our case, dirofilarias usually affect adult females, and microfilaremia, eosinophilia, and immunoglobulin E elevation are absent.^3^ Serology is not helpful; polymerase chain reaction has been suggested,^2^ but it is resource consuming and not widely available. Because of the wide variety of the differential diagnoses, surgical excision is the most used technique to reach the diagnosis.^3–5^ However, histopathology has limitations because the nematode morphology may be altered by inflammatory responses or surgical manipulations,^4^ as in our case.

To date, scarce data are available about ultrasonography of dirofilarial nodules; doppler imaging may also be helpful in the differential diagnosis.^4^ Older lesions present calcification and fibrosis,^5^ whereas younger nodules may show a weakly moving filiform worm (see Supplemental Video 1).^4^ In cases of surgical difficulty in reaching sites and controindications to diethylcarbamazine, ultrasonography should be considered for a watch and wait strategy. Physicians should be aware of this possibility to prompt the best diagnostic workup to avoid patient anxiety and invasive or unnecessary investigations.

## Supplementary Material

Supplemental Video.
